# Global hotspots and trends in the application of neoadjuvant therapy for gastric cancer: a bibliometric analysis

**DOI:** 10.3389/fonc.2026.1795575

**Published:** 2026-03-25

**Authors:** Zhongke Wang, Lingzu Kong, Tian-Yu Jiang, Qi Zhang, Lu Chen, Jinying Zhao, Fuchun Wang

**Affiliations:** 1 Department of Acupuncture and Tuina, Changchun University of Traditional Chinese Medicine, Changchun, China; 2 College of Acupuncture-Moxibustion and Orthopedics, Hubei University of Chinese Medicine, Wuhan, China

**Keywords:** Citespace, gastric cancer, immunotherapy, neoadjuvant therapy, VOSviewer

## Abstract

**Objective:**

Neoadjuvant therapy (NAT) is increasingly being applied in gastric cancer (GC) and is recognized by international guidelines. This study aims to analyze the current status, hotspots, and research trends of NAT in GC through bibliometrics.

**Methods:**

Relevant publications were collected from Web of Science (SCI-Expanded) and PubMed. We utilized Citespace to analyze countries, institutions, and keywords, and employed Vosviewer to examine authors and their co-cited authors, as well as journals and their co-cited journals.

**Results:**

The analysis encompassed 2666 documents. The overall number of publications showed an upward trend. The top-producing countries, institutions, authors, and journals in this field were: The People’s Republic of China and Chinese Academy of Medical Sciences - Peking Union Medical College, Huang, Changming, and Annals of Surgical Oncology. The most frequently cited author and journal are Al-batran and the Journal of Clinical Oncology, respectively. Key research areas in this field include: diverse NAT strategies; open-label trials and survival outcomes; and technology empowerment. Potential future research trends encompass: deep learning applications; immune checkpoint inhibitors; nivolumab; and immunotherapy.

**Conclusion:**

We used bibliometrics to conduct a visual analysis of the application of NAT in GC. Research hotspots include diverse NAT strategies, open-label trials and survival outcomes, and technology empowerment.

## 1 Introduction

Gastric cancer (GC), the fifth most common cancer globally, is a worldwide public health issue and the fifth leading cause of cancer-related deaths. GC is the most commonly diagnosed cancer in eight of 33 countries (1). GC is most prevalent among men, with > 1.08 million new cases diagnosed annually, resulting in a significant disease burden and mortality rate (2). East Asia has the highest incidence of gastric cancer, followed by Eastern and Central Europe (2). Over 60 per cent of new gastric cancer cases occur in East Asia (3). East Asia outperforms Western countries in age-standardized five-year net survival rates (4). Although surgery is the primary treatment option for GC, the 5-year survival rate after resection alone ranges from 20% to 50% in western countries and ~70% in eastern countries (5, 6). Therapeutic approaches also differ between East and West. In the United States and Europe, neoadjuvant chemotherapy and adjuvant chemoradiotherapy are employed, whereas in Asia, adjuvant chemotherapy is the standard treatment (7).

Neoadjuvant therapy (NAT) was first proposed by Emil Frei III in 1982 (8). In recent years, NAT strategies for GC patients have been continuously refined and developed. NAT for GC has gained widespread recognition in international guidelines. In Western nations, the Medical Research Council published the three-phase MAGIC trial in 2006, which for the first time demonstrated that perioperative chemotherapy (epirubicin, cisplatin and infusion fluorouracil [ECF]) significantly outperformed surgery in improving progression-free survival rates. This study represented a landmark achievement (9, 10). This standard of care has been recommended for use in the NCCN gastric cancer guidelines for a decade (11). Based on this, the pivotal Phase III FLOT4 trial succeeded in establishing PLOT as the new standard of care (12). Key findings from the phase III PRODIGY study indicate that neoadjuvant docetaxel, oxaliplatin and S-1(DOS) followed by surgery and adjuvant S-1 confer benefit to Asian patients with resectable locally advanced gastric cancer (LAGC) (13). Another clinical study conducted in Asia, ATTRACTION-4 (14), demonstrated that nivolumab combined with oxaliplatin chemotherapy significantly improved progression-free survival in Asian patients with untreated, HER2-negative, unresectable advanced or recurrent gastric cancer or gastro-esophageal junction cancer. This marks nivolumab combined with oxaliplatin chemotherapy as a first-line treatment regimen. NAT can reduce tumor staging, improve surgical outcomes, and increase long-term survival rates (15). Recent findings from the PRODIGY study (16) indicated that neoadjuvant DOS chemotherapy improved overall survival outcomes in patients with LAGC, and it should be considered one of the standard treatments for Asian patients with LAGC.

Bibliometrics is a systematic scientific method for evaluating knowledge domains, offering opportunities to enhance the timeliness, accessibility, and reproducibility of literature research within academic fields (17). Bibliometric analysis can assist researchers in understanding recent developments and key areas within this frontier research domain, predicting future trends, and selecting research directions (18). Currently, numerous bibliometric studies are being applied within the fields of medical science and healthcare (19). This methodology is applied not only to basic medical research but also to clinical research (20, 21).Citespace and VosViewer can analyze collected literature data by country, institution, author, and keywords, facilitating the identification of hotspots and research trends within relevant fields. In recent years, research on NAT in GC has developed rapidly. However, there has been no bibliometric study examining the relationship between NAT and GC. Therefore, we used bibliometric analysis to examine the current state, hotspots, and research trends of NAT applications in GC, thereby providing direction for future studies.

## 2 Materials and methods

### 2.1 Data sources and detection strategy

All literature for this study was retrieved from the SCI-EXPANDED database within the Web of Science Core Collection and the local PubMed database at the Library of Changchun University of Chinese Medicine. The search period was from January 1, 2015, to November 5, 2025. We used “gastric cancer” and “neoadjuvant therapy” as search terms and supplemented them with subject headings from MESH (**Supplementary Table 1**).

#### 2.1.1 Inclusion criteria

The inclusion criteria are as follows: (i) The types of studies included in Web of Science were Articles and Review Articles; (ii) The types of studies included in PubMed comprised basic research, randomized controlled trials, clinical trials, and case reports; (iii) The language restriction was English; (iv) The time restriction was from 1 January 2015 to 5 November 2025.

#### 2.1.2 Exclusion criteria

The exclusion criteria are as follows: (i) Excluded types included Editorial Material, Meeting Abstract, Proceeding Paper, Letter, Correction, Retracted Publication, Early Access, and Book Chapters; (ii) Literature with low relevance to the subject.

#### 2.1.3 Researchers proceeded to screen further

Articles with low correlation with the Application of Neoadjuvant Therapy for Gastric Cancer.Lingzu Kong and Zhongke Wang first conducted independent screening, and after the disagreement, they consulted Zhao Jinying.

### 2.2 Data collection and analysis

In PubMed, we first selected the desired data, then clicked the “Send To” option, chose the citation manager, and saved the NBIB file to the specified directory. We initiated the conversion process, using the converted files in the output directory as input data files for CiteSpace, and naming them “download_*.txt”. In Web of Science, we exported in “Plain text file” format and named the file “download_*.txt”. Two files named “download_*.txt” were imported into Citespace (version 6.4.R1) and Vosviewer (version 1.6.20) for analysis. In CiteSpace, the parameter settings were: (1) time range: 2015–2025, with a slice of 1 year; (2) select node type once; and (3) set the Top-N option to 50. In the Citespace network knowledge map, nodes represent information units of analytical items. Node size indicates the frequency of occurrence for that analytical item. The color and width of the “growth rings” of the node reflect the temporal distribution and volume of occurrences for that item. The purple outer ring signifies that the analytical item possesses high centrality quality within the entire network. The thickness of connecting lines represents the strength of relationships between different analytical items.

## 3 Results

### 3.1 Literature screening status

**Figure 1:**
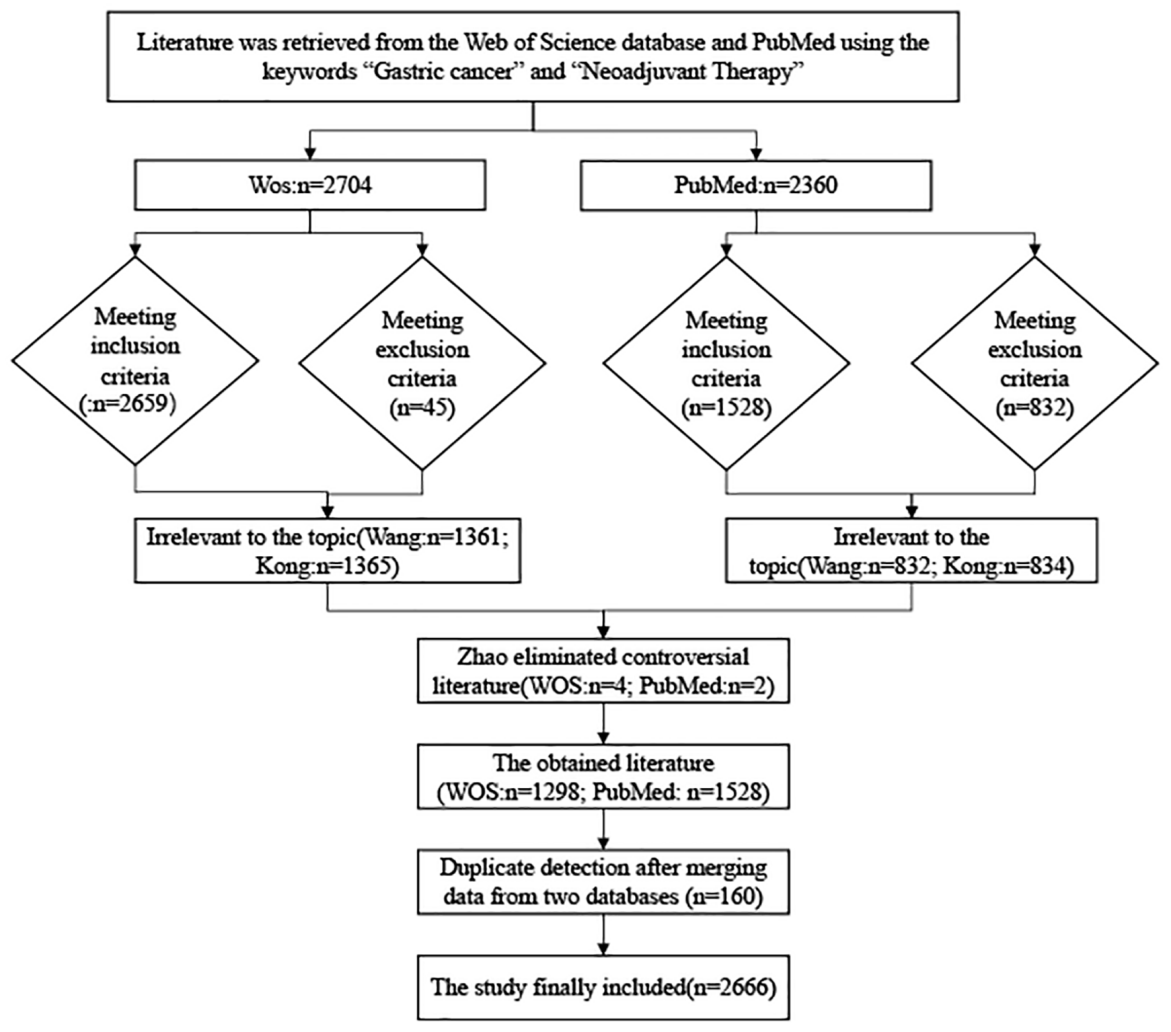
Flow diagram of the included papers.

The two researchers retrieved 2704 documents from Web of Science respectively; of which, they included 2256 original articles and 448 review articles. In terms of language, 2659 were in English, 34 in German, seven in Spanish, three in French, and one in Russian. They initially screened 2,659 documents. Wang excluded 1,361 documents deemed to have low relevance to the subject matter, while Kong excluded 1,365 documents for the same reason. Zhao was responsible for handling the four disputed documents. Zhao concluded that these four documents were also of low relevance to the subject matter and consequently removed them. We ultimately included 1,298 articles from Web of Science. The two researchers each included 2,360 articles from PubMed. Wang excluded 832 documents deemed irrelevant to the subject matter. Kong excluded 834 documents deemed irrelevant to the subject matter. Zhao identified an additional two documents as superfluous and irrelevant to the subject matter, and removed them. Finally, 1,528 articles were included from PubMed. We used the CiteSpace software to convert data from PubMed. We shall place the converted data alongside the filtered WOS data within a single folder for consolidation. We shall then employ CiteSpace software once more to conduct a duplicate detection check. After excluding 160 publications, we ultimately included 2,666 publications (**Figure 1**).

### 3.2 Analysis of annual publications

**Figure 2:**
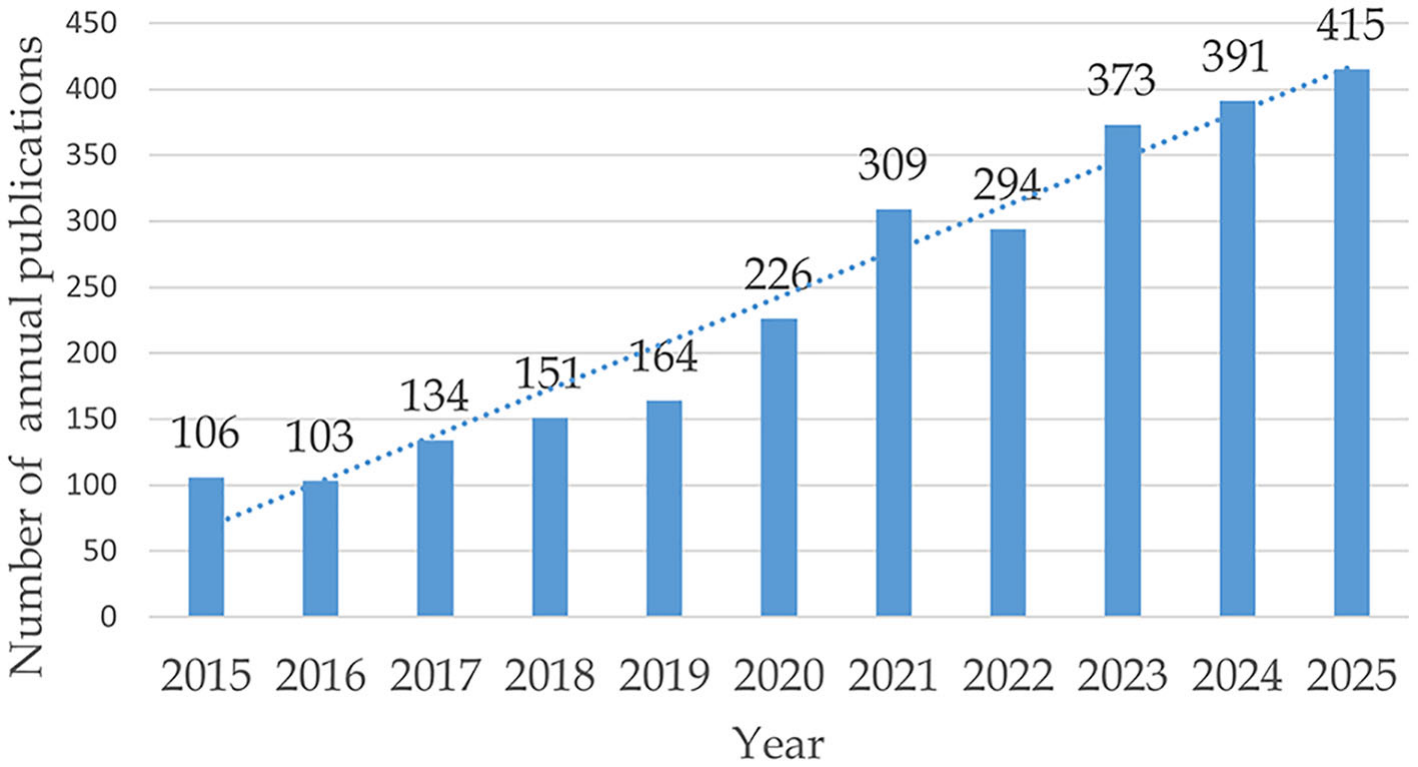
Analysis of annual publications.

We included 2666 documents. The number of annual publications showed an overall trend (**Figure 2**). From 2015 to 2021, the number of annual publications steadily increased, but in 2022, it showed a downward trend. After 2023, the growth rate of annual publications slowed considerably. By 2025, the total reached 415 publications. Based on data up to November 2025, the number of future publications is projected to continue growing.

### 3.3 Analysis of national cooperative network

**Figure 3:**
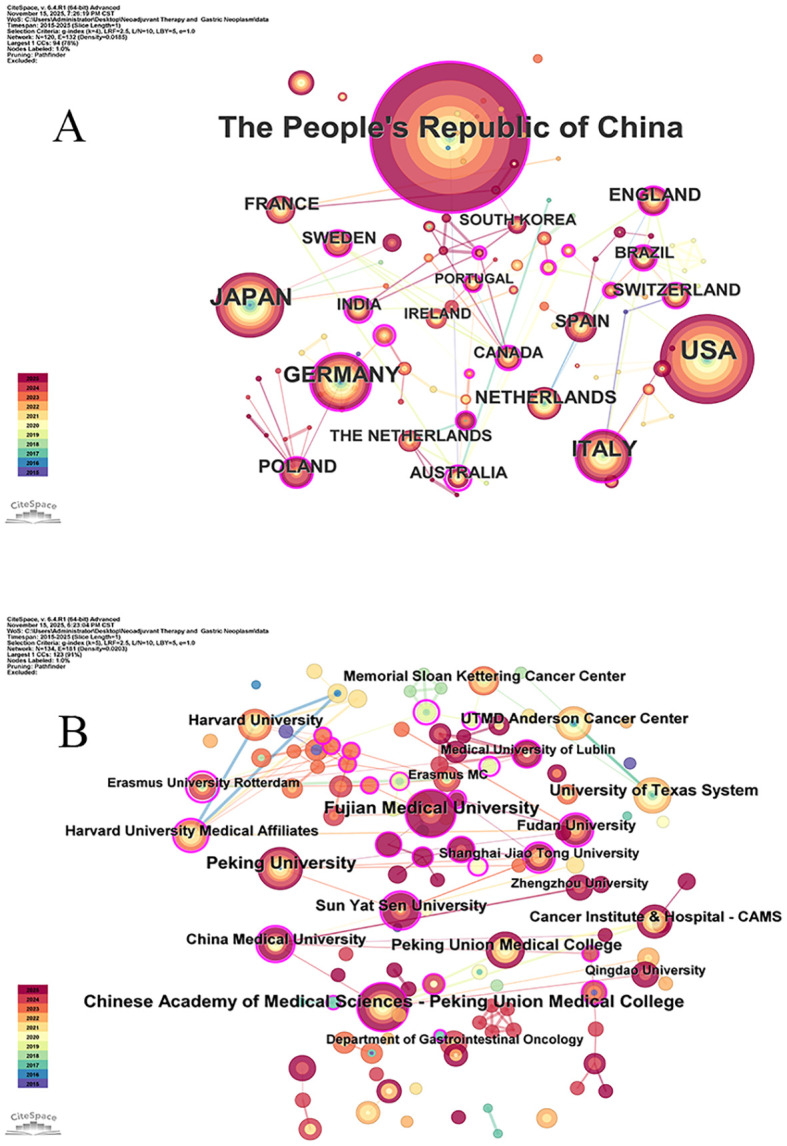
**(A)** Map of national cooperative networks; **(B)** Map of institutional cooperative networks.

A total of 120 countries and regions participated in research in this field (**Figure 3A**). **Table 1** lists the top five countries and regions by publications and centrality. The highest number of publications originated from China (n = 1185), followed by the USA (n = 461), Japan (n = 146), Germany (n = 181), and Italy (n = 166). In terms of centrality, these countries ranked as follows: Australia (0.61), Malaysia (0.46), India (0.43), Austria (0.42), and Czech Republic (0.37).

**Table 1:** Top five productive countries.

Rank	Publications	Country/region	Rank	Centrality	Country/region
1	1185	China	1	0.61	Australia
2	461	Usa	2	0.46	Malaysia
3	246	Japan	3	0.43	India
4	181	Germany	4	0.42	Austria
5	166	Italy	5	0.37	Czech Republic

### 3.4 Analysis of institutional collaboration network

A total of 134 institutions were engaged in research in this field (**Figure 3B**). **Table 2** lists the top five institutions by publication volume. Peking Union Medical College Hospital leads with the highest number of research outputs (n = 47), followed by Fujian Medical University (n = 45), Peking University (n = 43), University of Texas System (n = 33), and Sun Yat-sen University (n = 27). In terms of centrality (**Table 3**), the top-ranked institutions are Erasmus University (0.92), Shanghai Jiao Tong University (0.53), Harvard Medical School (0.49), Fudan University (0.46), and Sun Yat-sen University (0.45). Notably, all five leading institutions exhibit centrality scores exceeding 0.1.

**Table 2:** Top five productive institutions.

Rank	Publications	Institution
1	47	Chinese Academy of Medical Sciences - Peking Union Medical College
2	45	Fujian Medical University
3	43	Peking University
4	33	University of Texas System
5	27	Sun Yat Sen University

**Table 3:** Top five central institutions.

Rank	Centrality	Institution
1	0.92	Erasmus University Rotterdam
2	0.53	Shanghai Jiao Tong University
3	0.49	Harvard University Medical Affiliates
4	0.46	Fudan University
5	0.45	Sun Yat Sen University

### 3.5 Analysis of author and co-cited author

**Figure 4:**
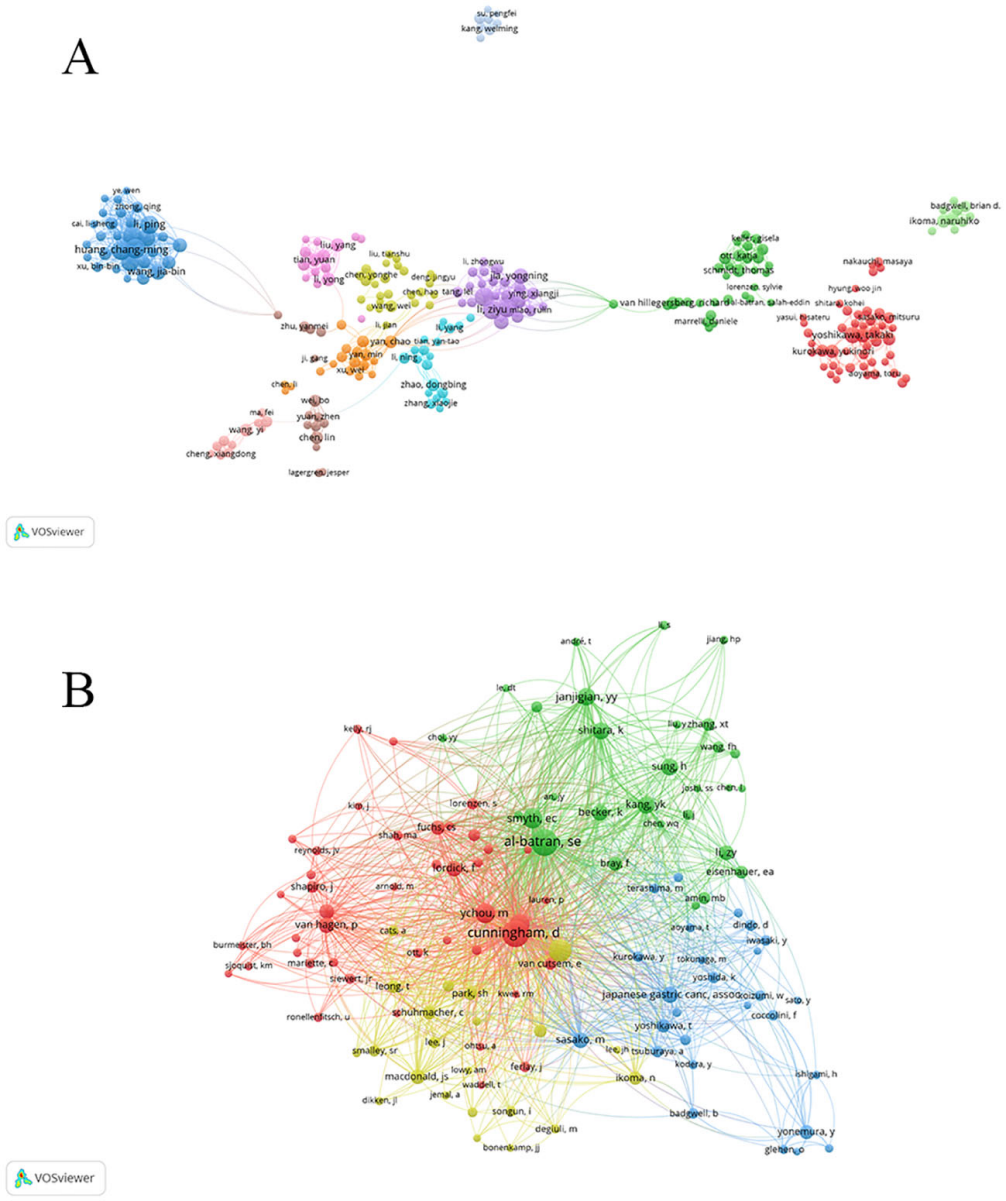
**(A)** Map of authors; **(B)** Map of co-cited authors.

We used Vosviewer to generate author collaboration networks and co-cited author networks. This included 11–186 authors and 2666 articles. **Figure 4A** displays authors with > 8 publications. Each node represents an author, with node size positively correlated to the author’s publication count. Links between nodes represent collaborative relationships, with line thickness indicating the strength of the collaboration. In the study on the application of NAT in GC, the first author is shown in **Table 4**. Ranked first is Huang, Changming, followed by Zheng, Chaohui, Li, Ziyu, Ji, Jiafu, and Xie, Jianwei.

**Table 4:** Top five authors and co-cited authors.

Rank	Publications	Author	Rank	Cited authors	Citations
1	63	Huang, changming	1	Al-batran, se	796
2	61	Zheng, chaohui	2	Cunningham, d	795
3	57	Li, ziyu	3	Ajani, ja	547
4	56	Ji, jiafu	4	Bang, yj	407
5	56	Xie, jianwei	5	Ychou, m	402

The total number of authors cited was 18 212, with 47 authors cited > 100 times. **Figure 4B** shows authors with citation frequencies > 45. **Table 4** lists the top five most frequently cited authors. Among them, Al-batran, SE was the most frequently cited author (796 citations), followed by Cunningham, D (795 citations), Ajani, JA (547 citations), Bang, YJ (407 citations), and Ychou, M (402 citations).

### 3.6 Analysis of journal and co-cited journal

**Figure 5:**
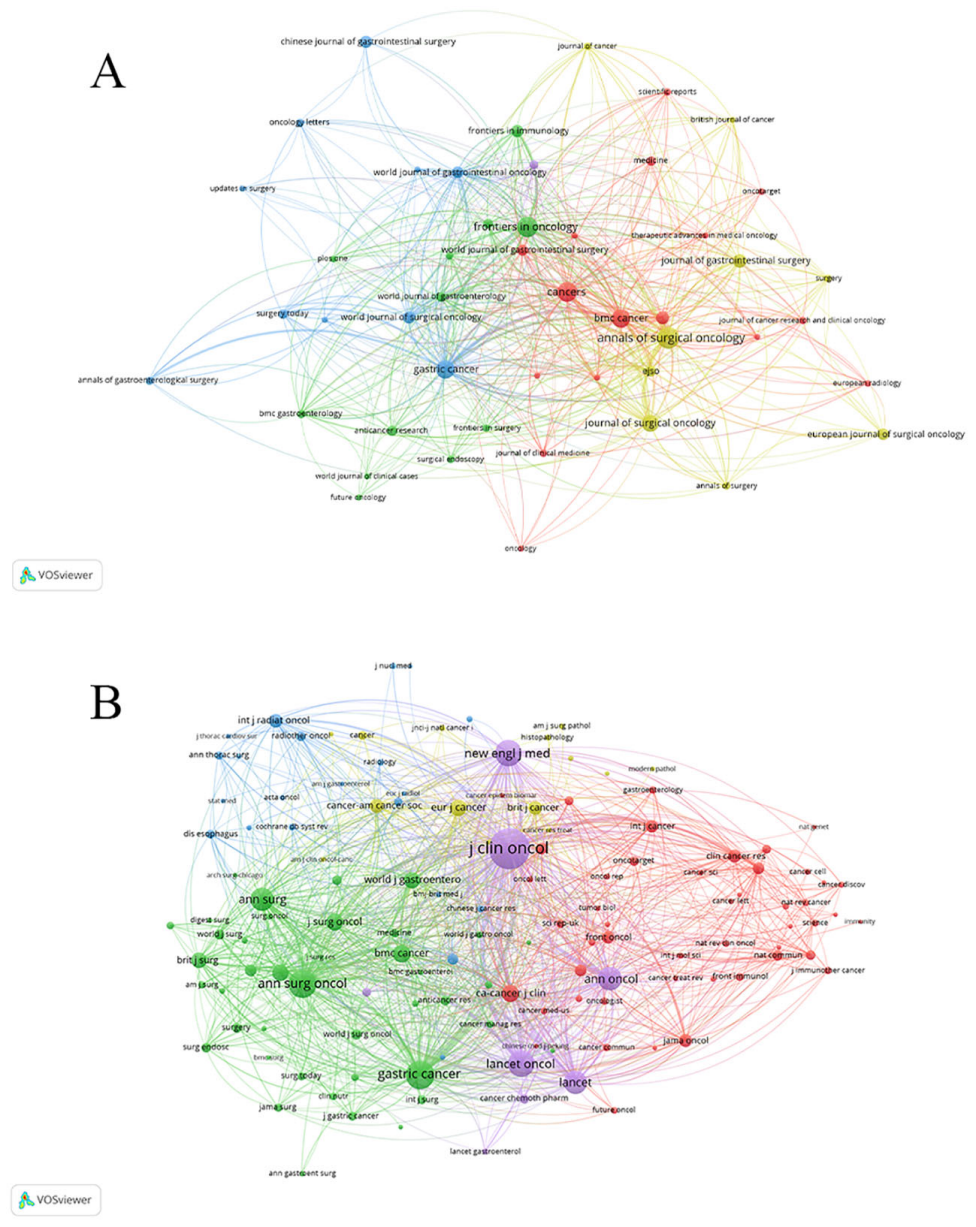
**(A)** Map of journals; **(B)** Map of co-cited journals.

We used Vosviewer to generate the journal and co-cited journals. Publications on the application of NAT in GC have been published in 502 journals. **Figure 5A** shows journals appearing > 10 times. **Table 5** presents the top 10 most popular journals in this field. Among them, three journals have published > 100 articles. They are: Annals of Surgical Oncology with the highest number of publications (n = 128), followed by Frontiers in Oncology (n = 110), and Cancers (n = 102). According to the 2025 Journal Citation Reports, five journals were classified as Q1, three journals were classified as Q2, one journal was classified as Q3, while Gan to Kagaku Ryoho Cancer & Chemotherapy was not classified. The journal with the highest impact factor was Cancers (42.5), while all others had impact factors < 6.

**Table 5:** Top 10 journals and co-cited journals.

Rank	Count	Journal	IF/JCR(2024-2025)	Rank	Co-citation	Co-cited Journal	IF/JCR(2024-2025)
1	128	Annals of Surgical Oncology	3.5/Q1	1	4644	Journal of Clinical Oncology	41.9/Q1
2	110	Frontiers in Oncology	3.3/Q2	2	2353	Gastric Cancer	5.1/Q1
3	102	Cancers	42.5/Q1	3	2175	Annals of Surgical Oncology	3.5/Q1
4	90	BMC Cancer	3.4/Q2	4	1998	Lancet Oncology	35.9/Q1
5	89	Gastric Cancer	5.1/Q1	5	1971	New England Journal of Medicine	78.5/Q1
6	80	Journal of Surgical Oncology	1.9/Q2	6	1588	Annals of Oncology	65.4/Q1
7	58	Gan to Kagaku Ryoho. Cancer & Chemotherapy	/	7	1564	Annals of Surgery	6.4/Q1
8	53	Journal of Gastrointestinal Surgery	2.4/Q1	8	1517	Lancet	88.5/Q1
9	47	Journal of Gastrointestinal Oncology	2/Q3	9	897	Bmc Cancer	3.4/Q1
10	43	Frontiers in Immunology	5.9/Q1	10	821	Journal of Surgical Oncology	1.9/Q2

**Figure 5B** shows journals with > 60 citations. **Table 5** lists the top 10 journals by citation frequency. Three journals received > 2000 citations. The most frequently cited journal was Journal of Clinical Oncology (4644), followed by Gastric Cancer (2353), and Annals of Surgical Oncology (2175). Nine of the journals with total citations were in Q1, while only one was in Q2. Five journals had an impact factor > 30. The highest-ranked journal was The Lancet (IF = 88.5), followed by New England Journal of Medicine (IF = 78.5), Annals of Oncology (IF = 65.4), Journal of Clinical Oncology (IF = 41.9), and Lancet Oncology (IF = 35.9). The impact factors of all other journals were < 7.

### 3.7 Analysis of keyword co-occurrence

**Figure 6:**
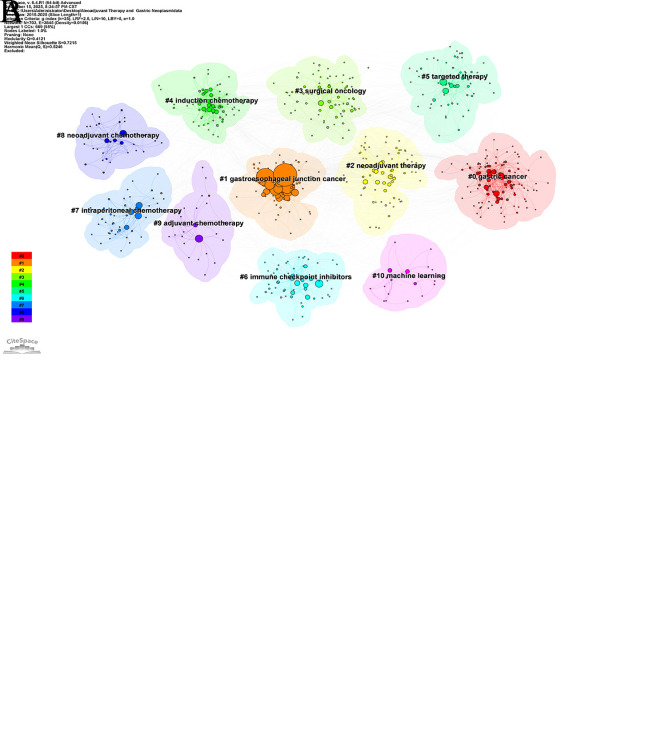
**(A)** Map of co-occurring keywords; **(B)** Map of keyword clustering.

The co-occurrence network visualization analysis diagram comprised 703 nodes and 3845 links, with a network density of 0.0156 (**Figure 6A**). As shown in **Table 6**, the top 10 keywords were as follows: gastric cancer (1585), neoadjuvant chemotherapy (919), neoadjuvant therapy (551), surgery (439), perioperative chemotherapy (395), adenocarcinoma (338), survival (254), open label (251), cancer (221), and chemoradiotherapy (207).

**Table 6:** Top 10 keywords.

Rank	Frequency	Keywords
1	1585	Gastric cancer
2	919	Neoadjuvant chemotherapy
3	551	Neoadjuvant therapy
4	439	Surgery
5	395	Perioperative chemotherapy
6	338	Adenocarcinoma
7	254	Survival
8	251	Open label
9	211	Cancer
10	207	Chemoradiotherapy

### 3.8 Analysis of keyword clustering

The “K”-cluster-LSI algorithm was implemented to examine the keywords in this field of research. **Figure 6B** indicates that the modularity Q-value exceeded the threshold of 0.3, reaching 0.4121. This demonstrated a significant clustering structure. Additionally, the average contour value of the clustering was 0.7215, indicating that the clustering results were reasonable and reliable. This study yielded 11 clustered terms: #0 gastric cancer, #1 gastroesophageal junction cancer, #2 neoadjuvant therapy, #3 surgical oncology, #4 induction chemotherapy, #5 targeted therapy, #6 immune checkpoint inhibitors, #7intraperitoneal chemotherapy, #8 neoadjuvant chemotherapy, #9 adjuvant chemotherapy, #10 machine learning. #0 and #1 relate to GC type; #2, #4, #8, and #9 pertained to treatment strategy; #3, #5, #6, and #7 concerned specific treatment modalities; and #10 related to research methodology.

### 3.9 Analysis of keywords with the strongest citation bursts

**Figure 7:**
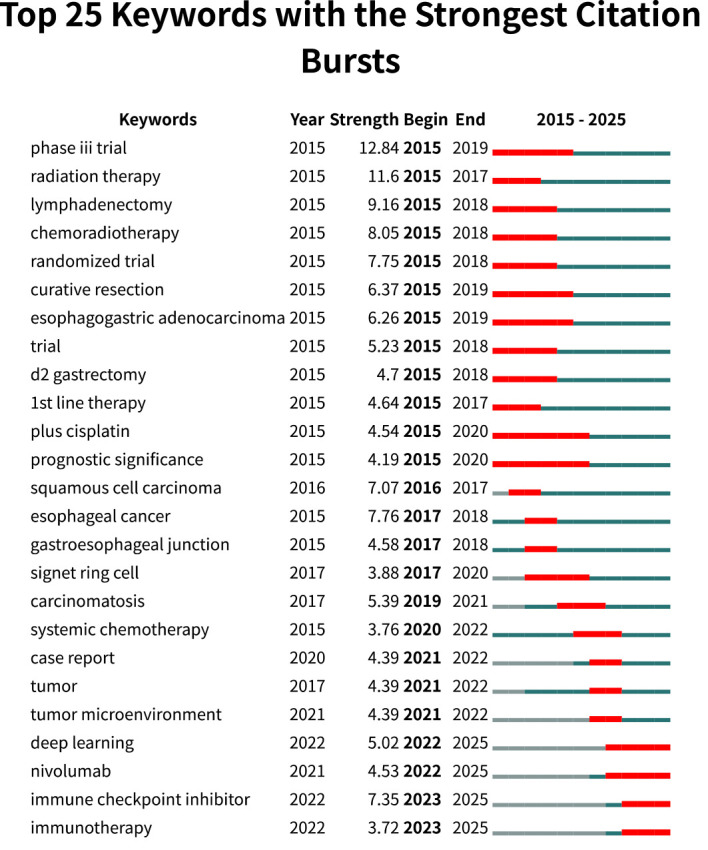
Top 25 of keywords with the strongest citation bursts.

Through salience analysis of keywords from the screened literature, we identified 25 keywords clustering (**Figure 7**). The red bars represent the start, duration, and end times of the burst. Phase 3 trials emerged earliest, exhibited the highest burst strength (12.84), and persisted for a longer duration. The keywords “plus cisplatin” and “prognostic significance” appeared earliest and persisted the longest (2015–2020). Since 2022, keywords such as deep learning, nivolumab, immune checkpoint inhibitor, and immunotherapy have garnered significant attention. This indicates that these keywords will become hotspots and trends in future research.

## 4 Discussion

### 4.1 General information

This study used Citespace and Vosviewer software to conduct a visual analysis of the application of NAT in GC. We included 2666 literature sources. Excel was used to analyze publication volume trends, and Citespace to examine country, institution, keyword co-occurrence, and keyword clustering. Vosviewer was utilized to analyze authors and co-cited authors, as well as journals and co-cited journals. The aim was to explore the current state, hotspots, and trends in the application of NAT within GC.

Research on the application of NAT has been extensive. The growth rate of publications was slow between 2015 and 2019 but accelerated significantly in 2020 and 2021. Publication output declined in 2022 but remained higher than in 2020 and earlier years. The growth rate of publications has slowed since 2020, although the volume remains substantial. We have only retrieved data up to November 5, 2025, but the current publication volume is already at its highest. We predict that the publication volume in 2025 will continue to increase to a new peak.

Research on the application of NAT in GC has been conducted in 120 countries or regions, which may be related to GC being the fifth most common malignant tumor. China ranked first in the number of publications, with ~2.5 times more publications than the second-ranked country. Australia had the highest centrality, indicating that Australian articles had the broadest influence. An international phase III clinical trial conducted in Australia demonstrated that neoadjuvant therapy increased overall survival rates in gastric cancer patients, adding to the evidence for perioperative chemotherapy (22). Additionally, Australia participated in the 4th St. Gallen EORTC Gastrointestinal Cancer Conference, addressing several contentious issues concerning neoadjuvant therapies for gastric cancer (23). The top five countries by number of publications were China, Japan, USA, Germany, and Italy. This may be related to the incidence and mortality rates of GC. The region with the highest age-standardized rate (ASR) globally was East Asia, followed by Europe (2). Although the USA is considered a country with low incidence rates, GC has a higher incidence among some minority populations (24). Visual analysis of the national cooperation network revealed numerous but thin lines connecting countries. This indicated that while cooperation existed between nations, it was not particularly close.

The institution with the highest number of publications was the Chinese Academy of Medical Sciences - Peking Union Medical College. Four of the top five institutions by publication volume were from China, consistent with the fact that China was the country with the highest publication volume. The institution with the highest centrality was Erasmus University Rotterdam, indicating that its publications exerted the greatest influence. Erasmus University Rotterdam’s participation in clinical research on neoadjuvant therapy for Dutch patients with resectable gastric cancer played a significant role in formulating international interim treatment adjustment guidelines (25). Erasmus University Rotterdam participated in a multicenter European clinical study (26), which demonstrated that local tumor control is of paramount importance and can be achieved through neoadjuvant chemotherapy combined with extended lymphadenectomy or limited lymphadenectomy with chemotherapy.

The top five authors by publication volume were all from China. Among them, Huang, Changming, Zheng, Chaohui, and Xie were affiliated with Fujian Medical University. They belonged to the same research team and maintained close collaboration. Their research in this field focused on the prognostic survival outcomes of NAT for GC. They used a novel staging system based on residual tumor burden, which significantly outperformed ypTNM (postneoadjuvant therapy pathologic tumor, node, metastasis, ypTNM) staging in improving the accuracy of prognostic prediction for patients following NAT for GC (27). Li, Ziyu, and Ji were affiliated with Peking University. They maintained close collaboration, with their research in this field focusing on laparoscopic gastrectomy. Their study demonstrated that in advanced GC following NAT, laparoscopic total gastrectomy with D2 lymph node dissection performed by an experienced surgical team yielded 3-year overall survival and disease-free survival rates comparable to those achieved with open total gastrectomy (28). Among co-cited authors, Al-batran had the highest citation frequency. His research primarily focused on multimodal therapy for locally advanced GC. Next was Cunningham, whose research focused on perioperative chemotherapy for GC. Ajani, ranked third, focused his research primarily on the survival rates of new adjuvant therapies for GC. Bang, ranked fourth, focused his research on the efficacy and safety of NAT for GC. Ychou, ranked fifth, and his research focused on survival rates for GC following NAT.

The journal with the highest number of publications was Annals of Surgical Oncology, which also ranked third in citation frequency. This indicated that the journal provided substantial support for research in this field and achieved significant dissemination impact. The top three journals in terms of publication volume have published a large number of articles, each exceeding 100 papers, which indicates that research in this field has gained significant recognition. Among the top 10 journals by publication volume, five were classified as Q1, three as Q2, and one as Q3. This indicates that research in this field is profound and in demand, and capable of being published in high-quality journals. Gan to Kagaku Ryoho Cancer & Chemotherapy is the official journal of the Japanese Gastric Cancer Association. Although not indexed in Science Citation Index, it maintains a high publication volume, which indicates Japan’s advanced research in this field. In terms of impact factor, Cancers had the highest impact factor, > 40. The impact factors of the remaining journals were all < 6, indicating that the influence of these journals needs to be enhanced. Among the top 10 most cited journals, Journal of Clinical Oncology had the highest citation count with an impact factor of 41.9, indicating that this journal publishes high-quality articles in the field and wields significant influence. Nine co-cited journals belonged to Q1, while only one co-cited journal belonged to Q2. Five journals had an impact factor > 30, four between 3 and 7, and only one < 3. This indicates that research in this field exerts high-quality influence.

### 4.2 Research hotspots

Through analysis of keyword and keyword clustering, current research hotspots can be identified. The primary focus areas are diverse NAT strategies, open label and survival, and technology empowerment.

#### 4.2.1 Diverse NAT strategies

NAT for GC treatment has been rapidly incorporated into international and western guidelines (29). NAT encompasses conventional preoperative chemotherapy, chemoradiotherapy, neoadjuvant chemotherapy, neoadjuvant chemotherapy combined with targeted therapy, molecular targeted therapy, and immunotherapy (30, 31). This therapy can reduce tumor staging, eliminate micrometastases, improve tolerability, increase the likelihood of curative resection, enhance resection rates in Ro patients, and ultimately prolong overall survival (32). With the ongoing advancement of NAT, numerous clinical trials and novel therapeutic concepts have emerged. Treatment strategies have evolved from conventional NCT to include neoadjuvant immunotherapy plus chemotherapy (NICT), NCT plus targeted therapy (NCTT), NICT plus targeted therapy (NICTT), and other innovative therapies (33). Recently, the concept of total neoadjuvant therapy (TNT) has been proposed. The most common sequence for TNT is induction chemotherapy, followed by chemotherapy (34).

#### 4.2.2 Open-label trials and survival outcomes

Open label refers to a trial in which everyone involved in an uncontrolled or single-arm study knows what treatment the subjects are receiving (35). Although open-label studies carry the risk of subjective judgment and are less reliable than results from randomized double-blind trials, they remain common in oncology randomized controlled trials (36). It is estimated that the therapeutic efficacy of treatments lacking double-blind trials is higher than that of double-blind trials (37). In this study, an open-label randomized controlled trial was commonly used to observe the survival outcomes of patients following NAT treatment for GC. Zheng et al. (38) conducted a single-arm, open-label, phase 2 clinical trial confirming the efficacy and safety of apatinib plus NCT in locally advanced gastric adenocarcinoma. Tian et al. (39) conducted an open-label, randomized controlled trial confirming that NCT can increase the pathological complete response rate and improve overall survival and disease-free survival in patients under 60 years of age with advanced GC.

#### 4.2.3 Technology empowerment

Machine learning is an emerging field in medicine that requires significant resources to apply computer science and statistics to medical practice (40). In the field of precision medicine, machine learning has been extensively applied in cancer research, where it is used to develop diagnostic, prognostic, and predictive tools from single-omics data (41). The application of machine learning in GC diagnosis and management is becoming increasingly widespread. A systematic review and meta-analysis revealed (42) that machine learning based on radiomics can predict response to neoadjuvant chemotherapy and survival rates in patients with locally advanced GC. Huang et al. (43) utilized machine learning techniques to predict postoperative survival rates and recurrence patterns in locally advanced GC following NAT.

### 4.3 Frontiers in research

The prominence of certain keywords reflects current research trends to some extent. Keywords such as deep learning, nivolumab, immune checkpoint inhibitor, and immunotherapy have remained popular to this day, indicating future research directions.

#### 4.3.1 Deep learning applications

Deep learning is a category of machine learning and AI methods based on multilayer artificial neural networks, which have been demonstrated to outperform traditional supervised learning approaches in numerous applications (44). The increasing prevalence of deep learning in the medical field, coupled with the availability of highly characterized cancer datasets, has accelerated its application in analyzing the complex biology of cancer. Specific applications of deep learning in oncology include cancer origin detection, molecular subtype identification, prognosis and survival prediction, histological inference of genomic traits, tumor microenvironment analysis, and future applications in spatial transcriptomics, metagenomics, and pharmacogenomics (45). Deep learning significantly reduces the difficulty of feature extraction by automatically learning representations of key disease characteristics from medical images (46). Deep learning holds significant potential for predicting response to neoadjuvant therapy, pathological outcomes, survival rates, and prognosis in gastric cancer patients. Research by Qiu et al. (47) indicated that the co-attention triple-guided spatial Mamba was a multitask deep learning model trained on longitudinal computed tomography (CT) images of locally advanced GC patients undergoing NCT. It can accurately predict lymph node metastasis and overall survival rates. Research by Zhang et al. (46) demonstrated that CT-based deep learning models demonstrated strong performance in predicting drug resistance among patients with locally advanced gynecologic cancer undergoing NCT. Research by Zheng et al. (48) showed that deep learning models based on transformers can predict lymph node metastasis and survival outcomes in patients with locally advanced gynecologic cancer who receive NCT, offering promise for personalized treatment. However, deep learning still has limitations. When confronted with specific clinical problems and tumor types, its application becomes restricted (49). Further research is required to clarify the features within tumors and their precise biological significance through deep learning (50).

#### 4.3.2 Immunotherapy: immune checkpoint inhibitors and nivolumab

Immunotherapy has emerged as an effective treatment for GC following surgery, chemotherapy, radiotherapy, and targeted therapy, and represents one of the breakthroughs in cancer treatment (51). Immunotherapy has ushered in a new era in cancer treatment, and cancer immunotherapy continues to evolve. The clinical objective of cancer immunotherapy is to activate the host immune system, enabling it to provide passive or active immunity against malignant tumors (52). In recent years, immunotherapy has demonstrated remarkable efficacy in GC. Combining immunotherapy with chemotherapy, targeted therapy and other approaches in the context of neoadjuvant treatment for gastric cancer not only enhances patients’ therapeutic response but also effectively prolongs their survival (53). Neoadjuvant immunotherapy and neoadjuvant immunotherapy combined with chemotherapy have demonstrated potential in improving the pathological complete response rate and survival rates among gastric cancer patients (54). Immune checkpoint inhibitors have been developed and studied in both preclinical and clinical settings for GC (55). Yu et al. (56) conducted a systematic review and meta-analysis on the widespread application of programmed cell death protein (PD)-1 as an immune checkpoint inhibitor. They concluded that the combination of PD-1 inhibitors with NAT enhances the likelihood of achieving curative surgery and improves prognosis in patients with locally advanced GC. Furthermore, they predicted that PD-1 combined with NAT will emerge as the preferred NAT option for locally advanced GC. Ooki et al. (57) propose that mismatch repair deficiency/microsatellite instability-high serves as a robust predictive biomarker for immune checkpoint inhibitor therapy in GC, highlighting the potential of immune checkpoint inhibitors as a new adjuvant therapy. The latest breakthroughs in immune checkpoint inhibitors have ushered in a new era for cancer immunotherapy (58). Nivolumab is a PD-1 inhibitor, a monoclonal antibody that has been adopted as a novel approach for treating recurrent and advanced GC (55). In clinical trials, patients treated with nivolumab demonstrated longer survival rates compared to those receiving placebo or chemotherapy (59). Research by Hasegawa et al. (60) indicates that nivolumab as adjuvant therapy for patients with resectable GC demonstrates safety and induces major pathological responses in certain resectable GCs. Research by Kang et al. (14) demonstrated that nivolumab combined with oxaliplatin-based chemotherapy improved progression-free survival in Asian patients with untreated, HER2-negative, unresectable advanced or recurrent GC or gastroesophageal junction cancer. However, several unresolved issues remain. Firstly, it is currently unclear which neoadjuvant immunotherapy is most effective for treating gastric cancer (61). Secondly, the binding of PD-1 and PD-L1 suppresses the host’s anti-tumor immunity, leading to tumor immune escape (62). Finally, neoadjuvant immunotherapy may trigger safety concerns and adverse reactions (54).

## 5 Advantages and limitations

We combined bibliometric analysis with visual methods to systematically describe the current research status, hotspots, and evidence-based trends of NAT in GC. The study aimed to provide readers with a clear, structured overview of research progress and emerging trends in this field. However, this study still had several limitations. We utilized only two databases, Web of Science and PubMed, which may have resulted in an incomplete representation of the existing literature. This may exclude valuable research from other databases, thereby affecting the representativeness and comprehensiveness of the findings. Future work will enhance the comprehensiveness of research findings by incorporating additional databases (e.g. Scopus, Cochrane) or incorporating manual review. Due to the ongoing and dynamic nature of the database, publications released after the search date were excluded from the study. Furthermore, the software imposed limitations on data formats, preventing the simultaneous analysis of references and other related content. At present, only a preliminary overview of the current status and research trends in applying NAT to the field of garbage collection can be provided. Therefore, a comprehensive introduction to this field still requires further refinement.

## 6 Conclusion

To our knowledge, this is the first comprehensive quantitative bibliometric analysis of NAT applications in GC. This study analyzed factors including annual publications, countries, institutions, authors, journals, and keywords using Citespace and Vosviewer software. Key research areas have been identified: Diverse neoadjuvant therapy strategies; Open-label trials and survival outcomes; and Technology Empowerment. Concurrently, future research directions have been clarified: deep learning, immune checkpoint inhibitors, nivolumab, and immunotherapy.

## Data Availability

The original contributions presented in the study are included in the article/**Supplementary Material**. Further inquiries can be directed to the corresponding authors.
