# Supplementary material for: Global hotspots and trends in the application of neoadjuvant therapy for gastric cancer: a bibliometric analysis
Source: Front Oncol. 2026 Mar 25;16:1795575. doi: 10.3389/fonc.2026.1795575 (PMC13056663; doi:10.3389/fonc.2026.1795575)
Supplement: Supplementary file 1 [file Table1.docx]

**Supplementary Tables**

Table 1 Top five productive countries

| Datebase | Search term |
| --- | --- |
| PubMed | #1 Stomach Neoplasms[Mesh] |
|  | #2 Neoplasm, Stomach[Title/Abstract] OR Stomach Neoplasm[Title/Abstract] OR Gastric Neoplasms[Title/Abstract] OR Gastric Neoplasm[Title/Abstract] OR Neoplasm, Gastric[Title/Abstract] OR Neoplasms, Gastric[Title/Abstract] OR Neoplasms, Stomach[Title/Abstract] OR Cancer of Stomach[Title/Abstract] OR Stomach Cancers[Title/Abstract] OR Cancer of the Stomach[Title/Abstract] OR Gastric Cancer[Title/Abstract] OR Cancer, Gastric[Title/Abstract] OR Cancers, Gastric[Title/Abstract] OR Gastric Cancers[Title/Abstract] OR Stomach Cancer[Title/Abstract] OR Cancers, Stomach[Title/Abstract] OR Cancer, Stomach[Title/Abstract] OR Gastric Cancer, Familial Diffuse[Title/Abstract] |
|  | #3 #1 OR #2 |
|  | #4 Neoadjuvant Therapy[Mesh] |
|  | #5 Neoadjuvant Therapies[Title/Abstract] OR Therapy, Neoadjuvant[Title/Abstract] OR Neoadjuvant Treatment[Title/Abstract] OR Neoadjuvant Treatments[Title/Abstract] OR Treatment, Neoadjuvant[Title/Abstract] OR Neoadjuvant Chemotherapy[Title/Abstract] OR Chemotherapy, Neoadjuvant[Title/Abstract] OR Neoadjuvant Chemotherapies[Title/Abstract] OR Neoadjuvant Chemotherapy Treatment[Title/Abstract] OR Chemotherapy Treatment, Neoadjuvant[Title/Abstract] OR Neoadjuvant Chemotherapy Treatments[Title/Abstract] OR Treatment, Neoadjuvant Chemotherapy[Title/Abstract] OR Neoadjuvant Chemoradiotherapy[Title/Abstract] OR Chemoradiotherapy, Neoadjuvant[Title/Abstract] OR Neoadjuvant Chemoradiotherapies[Title/Abstract] OR Neoadjuvant Chemoradiation[Title/Abstract] OR Chemoradiation, Neoadjuvant[Title/Abstract] OR Neoadjuvant Chemoradiations[Title/Abstract] OR Neoadjuvant Chemoradiation Therapy[Title/Abstract] OR Chemoradiation Therapy, Neoadjuvant[Title/Abstract] OR Neoadjuvant Chemoradiation Therapies[Title/Abstract] OR Therapy, Neoadjuvant Chemoradiation[Title/Abstract] OR Neoadjuvant Chemoradiation Treatment[Title/Abstract] OR Chemoradiation Treatment, Neoadjuvant[Title/Abstract] OR Neoadjuvant Chemoradiation Treatments[Title/Abstract] OR Treatment, Neoadjuvant Chemoradiation[Title/Abstract] OR Neoadjuvant Systemic Therapy[Title/Abstract] OR Neoadjuvant Systemic Therapies[Title/Abstract] OR Systemic Therapy, Neoadjuvant[Title/Abstract] OR Therapy, Neoadjuvant Systemic[Title/Abstract] OR Neoadjuvant Systemic Treatment[Title/Abstract] OR Neoadjuvant Systemic Treatments[Title/Abstract] OR Systemic Treatment, Neoadjuvant[Title/Abstract] OR Treatment, Neoadjuvant Systemic[Title/Abstract] OR Neoadjuvant Radiotherapy[Title/Abstract] OR Neoadjuvant Radiotherapies[Title/Abstract] OR Radiotherapy, Neoadjuvant[Title/Abstract] OR Neoadjuvant Radiation[Title/Abstract] OR Neoadjuvant Radiations[Title/Abstract] OR Radiation, Neoadjuvant[Title/Abstract] OR Neoadjuvant Radiation Therapy[Title/Abstract] OR Neoadjuvant Radiation Therapies[Title/Abstract] OR Radiation Therapy, Neoadjuvant[Title/Abstract] OR Therapy, Neoadjuvant Radiation[Title/Abstract] OR Neoadjuvant Radiation Treatment[Title/Abstract] OR Neoadjuvant Radiation Treatments[Title/Abstract] OR Radiation Treatment, Neoadjuvant[Title/Abstract] OR Treatment, Neoadjuvant Radiation[Title/Abstract] |
|  | #6 #4 OR #5 |
|  | #7 #3 AND #6 |
| Web of science | #1 TS=(Stomach Neoplasms OR TS=Neoplasm, Stomach OR TS=Stomach Neoplasm OR TS=Gastric Neoplasms OR TS=Gastric Neoplasm OR TS=Neoplasm, Gastric OR TS=Neoplasms, Gastric OR TS=Neoplasms, Stomach OR TS=Cancer of Stomach OR TS=Stomach Cancers OR TS=Cancer of the Stomach OR TS=Gastric Cancer OR TS=Cancer, Gastric OR TS=Cancers, Gastric OR TS=Gastric Cancers OR TS=Stomach Cancer OR TS=Cancers, Stomach OR TS=Cancer, Stomach OR TS=Gastric Cancer, Familial Diffuse) |
|  | #2 TS=(Neoadjuvant Therapy OR TS=Neoadjuvant Therapies OR TS=Therapy, Neoadjuvant OR TS=Neoadjuvant Treatment OR TS=Neoadjuvant Treatments OR TS=Treatment, Neoadjuvant OR TS=Neoadjuvant Chemotherapy OR TS=Chemotherapy, Neoadjuvant OR TS=Neoadjuvant Chemotherapies OR TS=Neoadjuvant Chemotherapy Treatment OR TS=Chemotherapy Treatment, Neoadjuvant OR TS=Neoadjuvant Chemotherapy Treatments OR TS=Treatment, Neoadjuvant Chemotherapy OR TS=Neoadjuvant Chemoradiotherapy OR TS=Chemoradiotherapy, Neoadjuvant OR TS=Neoadjuvant Chemoradiotherapies OR TS=Neoadjuvant Chemoradiation OR TS=Chemoradiation, Neoadjuvant OR TS=Neoadjuvant Chemoradiations OR TS=Neoadjuvant Chemoradiation Therapy OR TS=Chemoradiation Therapy, Neoadjuvant OR TS=Neoadjuvant Chemoradiation Therapies OR TS=Therapy, Neoadjuvant Chemoradiation OR TS=Neoadjuvant Chemoradiation Treatment OR TS=Chemoradiation Treatment, Neoadjuvant OR TS=Neoadjuvant Chemoradiation Treatments OR TS=Treatment, Neoadjuvant Chemoradiation OR TS=Neoadjuvant Systemic Therapy OR TS=Neoadjuvant Systemic Therapies OR TS=Systemic Therapy, Neoadjuvant OR TS=Therapy, Neoadjuvant Systemic OR TS=Neoadjuvant Systemic Treatment OR TS=Neoadjuvant Systemic Treatments OR TS=Systemic Treatment, Neoadjuvant OR TS=Treatment, Neoadjuvant Systemic OR TS=Neoadjuvant Radiotherapy OR TS=Neoadjuvant Radiotherapies OR TS=Radiotherapy, Neoadjuvant OR TS=Neoadjuvant Radiation OR TS=Neoadjuvant Radiations OR TS=Radiation, Neoadjuvant OR TS=Neoadjuvant Radiation Therapy OR TS=Neoadjuvant Radiation Therapies OR TS=Radiation Therapy, Neoadjuvant OR TS=Therapy, Neoadjuvant Radiation OR TS=Neoadjuvant Radiation Treatment OR TS=Neoadjuvant Radiation Treatments OR TS=Radiation Treatment, Neoadjuvant OR TS=Treatment, Neoadjuvant Radiation) |
|  | #3 #1 AND #2 |
